# Novel Tet3 enzymes for next-generation epigenetic sequencing†

**DOI:** 10.1039/d4cb00315b

**Published:** 2025-03-10

**Authors:** Özge Simsir, Tobias Walter, Hanife Sahin, Thomas Carell, Sabine Schneider

**Affiliations:** a Department of Chemistry, Institute for Chemical Epigenetics, Ludwig-Maximilians Universität Munich Butenandtstr. 5-13 81377 Munich Germany thomas.carell@cup.lmu.de sabine.schneider@cup.lmu.de

## Abstract

Epigenetic regulation of gene expression is essential for cellular development and differentiation processes in higher eukaryotes. Modifications of cytosine, in particular 5-methylcytosine (5mdC), in DNA play a central role through impacting chromatin structure, repressing transposons, and regulating transcription. DNA methylation is actively installed by DNA methyltransferases and reversed through Tet-dioxygenase-mediated oxidation of 5mdC to 5-hydroxylmethylcytosine (5hmdC), 5-formylcytosine (5fdC), and 5-carboxycytosine (5cadC). It is crucial to understand the role of these epigenetic DNA modifications in cellular differentiation and developmental processes, as well as in disease state mapping and tracing of 5mdC and its oxidized forms. In bisulfite sequencing, which has been the benchmark for mapping 5mdC for the last few decades, degradation of the majority of genetic material occurs through harsh chemical treatment. Alternative sequencing methods often utilize Tet-enzyme-mediated oxidation of 5mdC to locate 5mdC and 5hmdC in genomic DNA. Herein, we report the development of novel Tet3-variants for oxidation-based bisulfite-free 5mdC- sequencing.

## Abstract 2

Epigenetic sequencing methods largely rely on oxidation of 5mdC by Tet-enzymes. We report the development and characterization of novel Tet3-variants for oxidation-based bisulfite-free 5mdC-sequencing.

## Graphical abstract

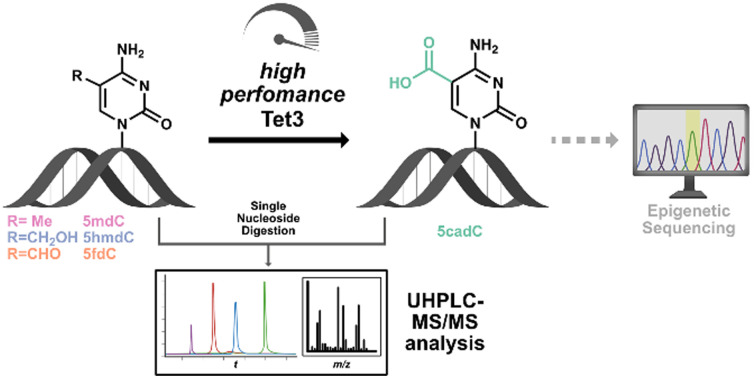

## Introduction

The family of Ten-eleven translocation (Tet) methylcytosine dioxygenases that oxidize 5-methyldeoxycytidine (mdC) in DNA, and thus lead to gene activation, was discovered in 2009.^1^ In humans, three Tet enzymes (Tet1–3) are known. They all use α-ketoglutarate (α-KG) and Fe(ii) to specifically oxidize 5mdC in the genome of vertebrates to 5-hydroxymethyl-dC (5hmdC), 5-formyl-dC (5fdC), and finally to 5-carboxy-dC (5cadC).^1–4^ This oxidation possibility has been recently employed to develop new sequencing methods for 5mdC and 5hmdC,^5–7^ providing milder reaction alternatives to the harsh conditions that occur in bisulfite sequencing.^8^ Mapping of 5mdC and 5hmdC is required to characterize the epigenetic state of genes, which is, for example, not only the basis for determining the age and differentiation of tissues but is also being considered for a procedure that will enable the detection of tumor DNA in blood samples in a process called liquid biopsy in the future.^9^

**Fig. 1:**
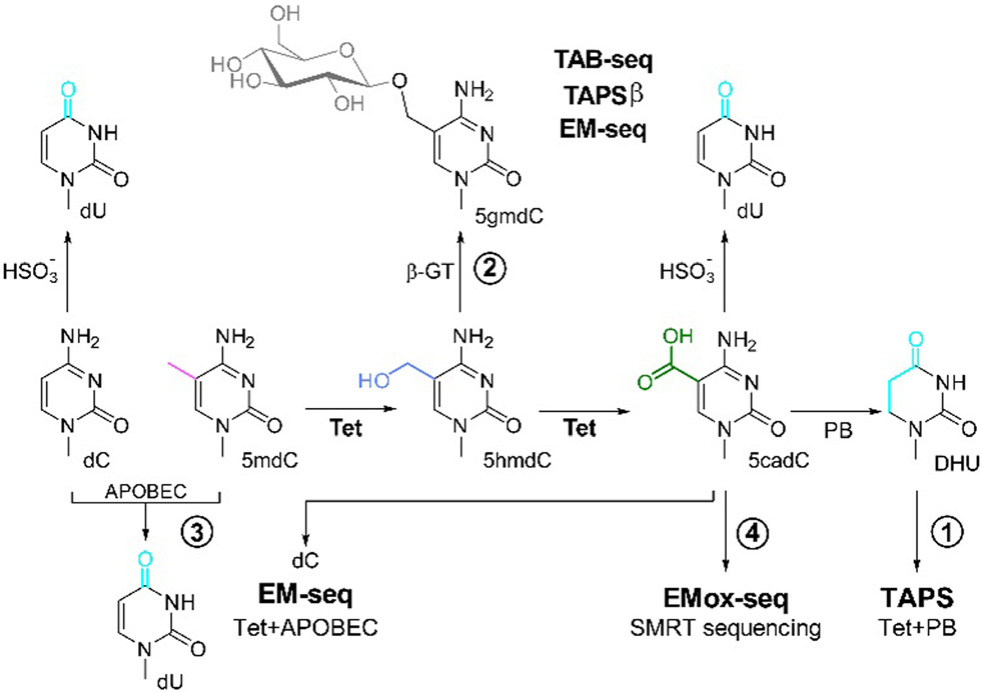
Overview of the current 5mdC and 5hmdC sequencing methods using Tet-enzymes for oxidation. (1) TAPS: Tet-mediated oxidation of 5mdC to 5cadC, followed by reduction by pyridine borane (PB) of the 5cadC to dihydrouracil (DHU), leading to the conversion of modified dC to dT in the sequencing reaction.^10^ (2) 5hmdC can be identified by protection of 5hmdC from the subsequent Tet-oxidation *via* glycosylation to 5gmdC using a β-glycosyltransferase (β-GT).^12^ This is followed by either conversion of dC and 5cadC with bisulfite to uracil (TAB-seq) or reduction of 5cadC by PB to DHU (TAPSβ).^11^ (3) EM-seq: protection of 5mdC from deamination by APOBEC through Tet-mediated oxidation of 5mdC *via* 5hmdC to 5cadC and simultaneous glycosylation of 5hmdC β-GT to 5gmdC. This results in the read-out of deaminated dC as dT, while 5cadC and 5gmdC are protected from APOBEC3-mediated deamination and read as dC in the subsequent sequencing.^7^ (4) EMox-seq: Tet-mediated, quantitative oxidation of 5mdC to 5cadC, which is directly read by SMRT sequencing.^6^

For the sequencing of 5mdC and 5hmdC, Tet-assisted pyridine borane sequencing (TAPS; method 1) was developed. 5mdC, 5hmdC, and also 5fdC (not shown) bases in the genome are oxidized with a Tet enzyme to 5cadC, which is followed by conversion of 5cadC with pyridine borane to give dihydrouridine (DHU, Fig. 1).^10^ For the identification of 5hmdC, 5hmdC can be protected from Tet-mediated-oxidation through glycosylation (=5gmdC) by β-glycosyltransferase (β-GT; method 2). Following protection of 5hmdC by glycosylation, 5mdC is oxidized to 5cadC by Tet treatment, and 5cadC is subsequently either converted with bisulfite to deoxyuridine (dU) (Tet-assisted bisulfite sequencing (TAB-seq))^11^ or it is again reduced with pyridine borane to DHU (TAPSβ) (Fig. 1).^12^ The position of dU/DHU is then decoded as “T” in the subsequent sequencing step, while 5gmdC is read as “C”. These steps provide positional information for 5mdC or 5hmdC in the genome.

In an alternative approach called enzymatic methyl sequencing (EM-Seq), 5mdC and 5hmdC are detected using two sets of enzymatic reactions. First, Tet2 oxidizes 5mdC *via* 5hmdC and 5fdC to 5cadC. 5hmdC is either present in the genetic material or generated as an intermediate product during 5mdC oxidation by Tet2, and is glycosylated by β-GT to 5gmdC. In the subsequent reaction, the remaining dC is deaminated to dU using apolipoprotein B mRNA editing enzymes (APOBEC, method 3), while 5cadC and 5gmdC are protected from deamination through APOBEC. The position of dU, corresponding to genomic dC, and 5mdC/5hmdC, are next identified by sequencing and comparison to a reference genomic sequence without the need to use bisulfite or pyridine borane (Fig. 1).^13,14^

For all these methods, modified Tet enzymes are required for the most efficient oxidation of 5mdC. Tet enzymes derived from Tet1 and Tet2 that have been described can efficiently oxidize 5mdC under the right conditions. However, achieving complete genome-wide oxidation remains challenging due to reaction conditions and enzyme turnover. For example, in the EM-Seq^13^ method, if a Tet protein was able to convert all 5mdCs into 5cadC, one might be able to remove the β-GT enzyme from the mix, which would simplify the procedure.

We recently reported a high-performance Tet-enzyme, based on mouse Tet3 (hpMmTet3), which was found to have superior oxidation capabilities.^6^ Moreover, we showed that following the quantitative oxidation of 5mdCs in genomic DNA to 5cadC, 5cadC can be directly read using PacBio high-fidelity, single molecule real time (SMRT) sequencing, without any further chemical or enzymatic treatment. This Tet3-based SMRT-sequencing method was termed Enzymatic Methyl oxidation sequencing (EMox-seq, Fig. 1, method 4).^6^ Herein, we report a more detailed and comparative study of different high-performance Tet (hpTet) enzymes, all based on Tet3, for improved 5mdC sequencing.

## Results and discussion

**Fig. 2:**
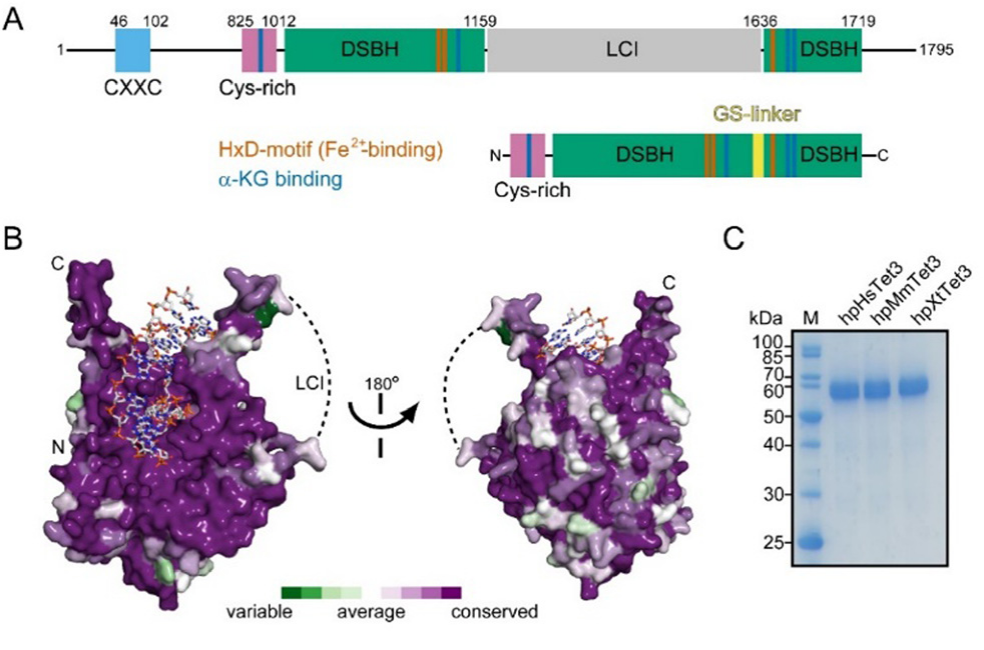
Schematic outline of the Tet3 proteins. (A) Domain structure of Tet3 and shortened Tet3-version (“hpTet3”) used in this study. The numbering corresponds to the full-length human Tet3. DSBH: double-stranded β-helix domain, LCI: low-complexity insert; α-KG: α-ketoglutarate. (B) Conservation of Tet3 homologs mapped onto the surface of the predicted structure of the catalytic domain (residues 821–1721, without LCI) of human Tet3 (Alphafold: AF-O43151-F1-model_v4). The LCI is indicated by the dashed line. For residues 1–825 and 1721–1795 as well as the LCI, no structure was predicted due to likely flexibility and/or disorder of these regions. (C) SDS-PAGE analysis of the purified hpTet3 proteins. *H. sapiens* hpHsTet3, *Mus musculus* hpMmTet3; *Xenopus tropicalis* hpXtTet3.

The Tet3-based constructs are shortened versions of Tet3 enzymes isolated from mouse, human, and western claw frog (Table S1, ESI†), sharing 84–95% sequence identity and 92–96% similarity (Fig. 2 and Fig. S1, Table S2, ESI†). In all three enzymes, we removed parts of the C- and N-termini as well as the low complexity inserts (LCI) interspacing the catalytic domain. In accordance with previous studies, the LCI was replaced by a short 15-mer Gly-Ser-linker.^6,15^ The residual proteins hpHsTet3, hpMmTet3, and hpXtTet3, depicted in Fig. 2A, start at the N-termini with a Cystein-rich domain, followed by a catalytically competent double-stranded β-helix (DSBH) domain. To facilitate purification, all proteins were equipped with an N-terminal Strep-tag II (Table S1, ESI†).

All three proteins were overexpressed in *Escherichia coli* and purified in two steps by first using a Streptactin XT column (affinity chromatography) followed by a second ion-exchange chromatography step (for details, see the Supporting Material and methods, ESI†). In all three cases, the result of the two-step purification protocol was proteins with a purity of >95% in yields of ≈2–3 mg L^−1^ of *E. coli* culture. Proteins with a purity of >99% were available with a yield of ≈1 mg L^−1^ culture (Fig. 2). For a sequence alignment of all three hpTet-enzymes and comparison with human Tet2, see Fig. S1 and S2 and Table S2 (ESI†). To study the oxidation capabilities, we first treated a 35-mer double-stranded oligonucleotide containing a single 5mdC within a CpG context (3 μmol) with one of the three hpTet3 constructs (5 μg) for 1 h at 37 °C. The protein was subsequently digested with proteinase K, and the DNA was then isolated, followed by total digestion with a mixture of commercially available enzymes (see the ESI†) to the nucleoside level.

**Fig. 3:**
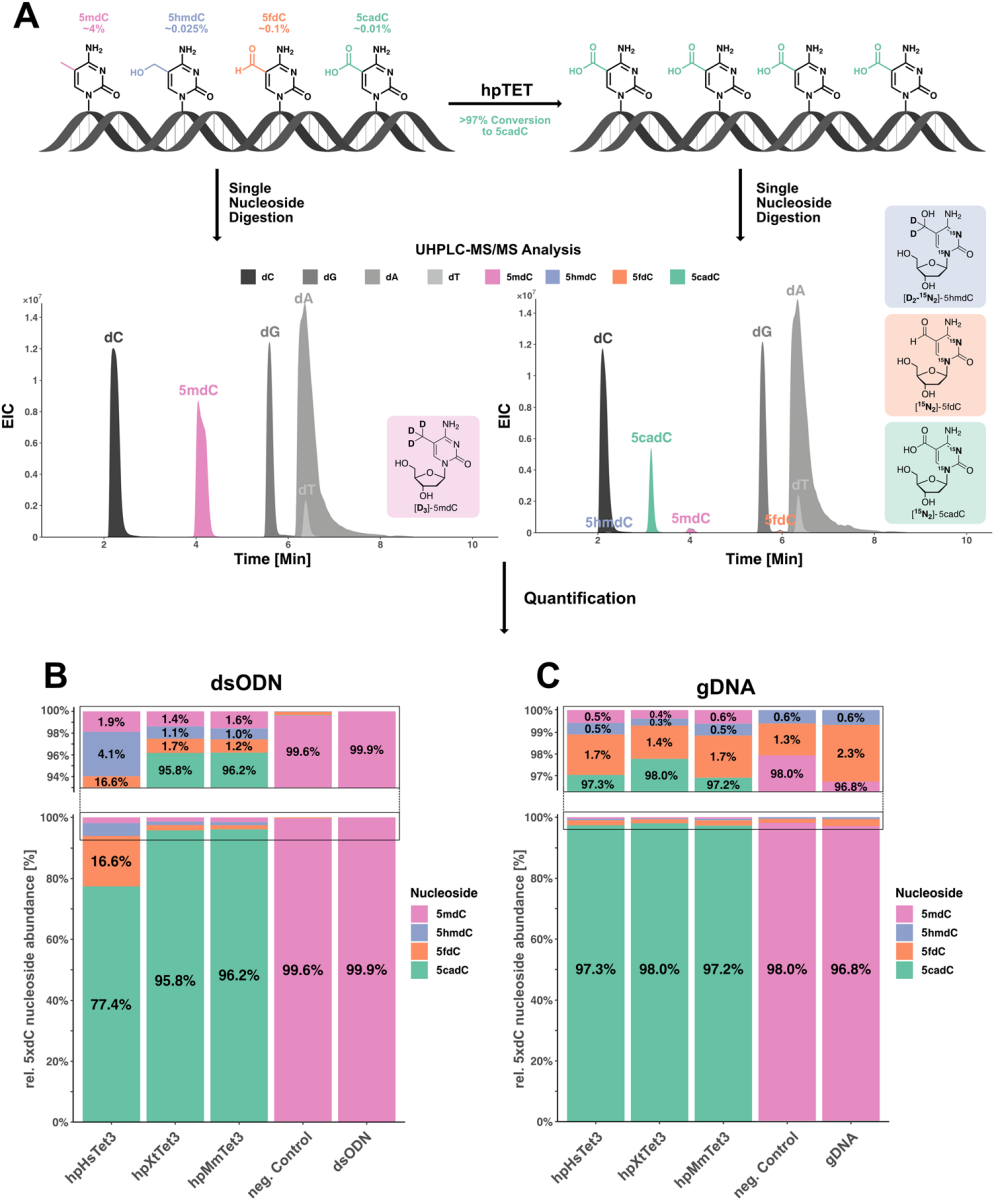
Oxidation of 5mdC by hpTet3-enzymes in synthetic and genomic DNA. (A) Principle of the LC-MS/MS-based quantification of 5mdC, 5hmdC, 5fdC, and 5cadC in hpTet3-treated dsODN/gDNA samples and structures of used isotopologs. (B) Plot of the relative percentage of the quantified modified cytidines in the double-stranded 35-mer and (C) HEK293T gDNA following enzymatic treatment. gDNA: isolated gDNA was digested, and dC and modified dC nucleosides were quantified. As a negative control, ODN or gDNA were incubated under Tet-oxidation conditions, but without the addition of hpTet-enzymes, followed by digestion and quantification.

We finally used our isotope dilution HPLC-coupled mass spectrometry method^16^ to perform an exact quantification of 5mdC, 5hmdC, 5fdC, and 5cadC (Fig. 3A). We subsequently noted that all three hpTet3 enzymes oxidized 5mdC with a yield of >98%. The best proteins for the conversion of 5mdC to 5cadC were the mouse and the xenopus hpTet3 enzymes, which converted 98.4% and 98.6% of the 5mdCs to higher oxidized compounds, respectively. Importantly, both enzymes generated 95.8% and 96.2% 5cadC (Fig. 3B and C).

We next investigated the oxidation of 5mdCs in genomic DNA by using genomic DNA isolated from HEK293T cells. For a control experiment, we digested the genomic DNA and measured the levels of 5mdC, 5hmdC, 5fdC, and 5cadC directly following isolation. We found that approximately 4% of all Cs are present as 5mdCs in this genomic material (Table S4, ESI†). We detected other oxidized nucleosides at levels of 0.025% 5hmdC, 0.1% 5fdC, and 0.01% 5cadC relative to the total amount of dC. These nucleosides, and in particular 5fdC, are likely oxidized lesions that formed mostly during DNA isolation and handling.^9,13^ Some present 5hmdC has certainly an epigenetic background. We next treated HEK293T gDNA (1 μg) with 10 μg of the different hpTet3 enzymes (1 h, 37 °C) and again isolated and digested the DNA.

The analysis using the isotope dilution method showed that with all three hpTet3 enzymes, >99.5% of 5mdC was converted (Fig. 3C). Again, 5cadC was by far the dominant reaction product, which formed in yields between 97–98%. In our hands, the enzyme that performed the highest conversion was hpXtTet3, with 5cadC formed at 98%, followed by the mouse and human variant at 97.2% and 97.3%, respectively (Fig. 3C), albeit hpXtTet3 appeared to be slightly less stable compared to hpMmTet3 and hpHmTet3 (Fig. S3, ESI†).

**Fig. 4:**
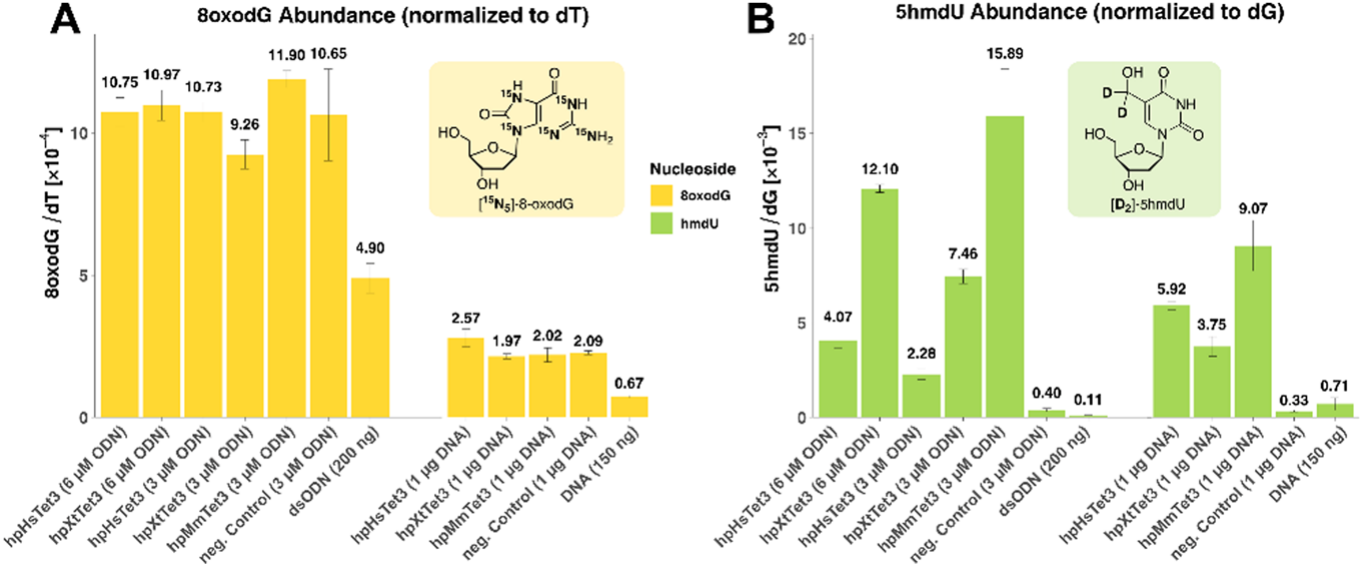
Analysis of the oxidation byproducts 8-oxodG and 5hmdU in synthetic and genomic DNA using the depicted isotope standards for quantification. (A) Amount of 8-oxodG per dT of ODN and gDNA, and (B) levels of 5hmdU per dG prior (control and gDNA/ODN) and following treatment with hpTet3 or solely with reaction buffer.

Oxidation of DNA, whether it enzymatically or chemically occurs, often generates oxidative lesions such as 8-oxodesoxyguanine (8-oxodG) and 5-hydroxymethlyuridine (5hmdU). A frequently overlooked problem is that many of these oxidized side products lead to sequencing errors. Particularly, 8-oxodG is decoded by most polymerases as a dG and dT nucleoside because it can pair with dA in its syn-conformation.^17,18^ To investigate and quantify the amount of oxidation byproducts, we used the 35-mer double-stranded oligonucleotide and genomic DNA to perform a deep mass spectrometry-based analysis of the oxidation byproducts using synthetic isotope standards, as depicted in Fig. 4. The obtained levels were normalized against dG (5hmdU) or dT (8-oxodG). We detected in the experiment that the levels of 8-oxodG and 5hmdU indeed increased after treatment of the DNA with the enzymes. For genomic DNA, we saw an increase in the 8-oxodG level by a factor of ≈2.5, from 0.07 8-oxodG/1000 dT to 0.2 8-oxodG/1000 dT (Fig. 4A). The 5hmdU levels increased by a factor of ≈20–45 from 0.2 5hmdU/1000 dG to between 4–9 5hmdU/1000 dG. Similar increases were detected for the 35-mer double strand (Fig. 4B).

This result is very surprising because the dG-nucleosides are typically those that are the most rapidly oxidized and are considered to be the prime targets for oxidative damage. However, the 8-oxodG levels only increased by a factor of approximately 2.5, while the 5hmdU levels increased 10-times stronger by a factor of about 20. This indicates that the oxidation could be enzyme-driven. It is possible that the hpTet3 enzymes might occasionally bind a dT instead of a 5mdC for oxidation to 5hmdU. However, we did not observe significant alterations of 5hmdU levels with higher enzyme concentrations (Fig. S4 and Table S6, ESI†). Importantly, the conversion of dT to 5hmdU does not change the coding potential and hence does not interfere with sequencing except for the above-mentioned TAPS and TAB-seq methods. Although this oxidation only adds 4–9 5hmdU/1000 dG, even when using higher enzyme concentrations (Fig. S4 and Table S6, ESI†) and is therefore a rather minor side fraction, it nevertheless needs to be taken into account.

**Fig. 5:**
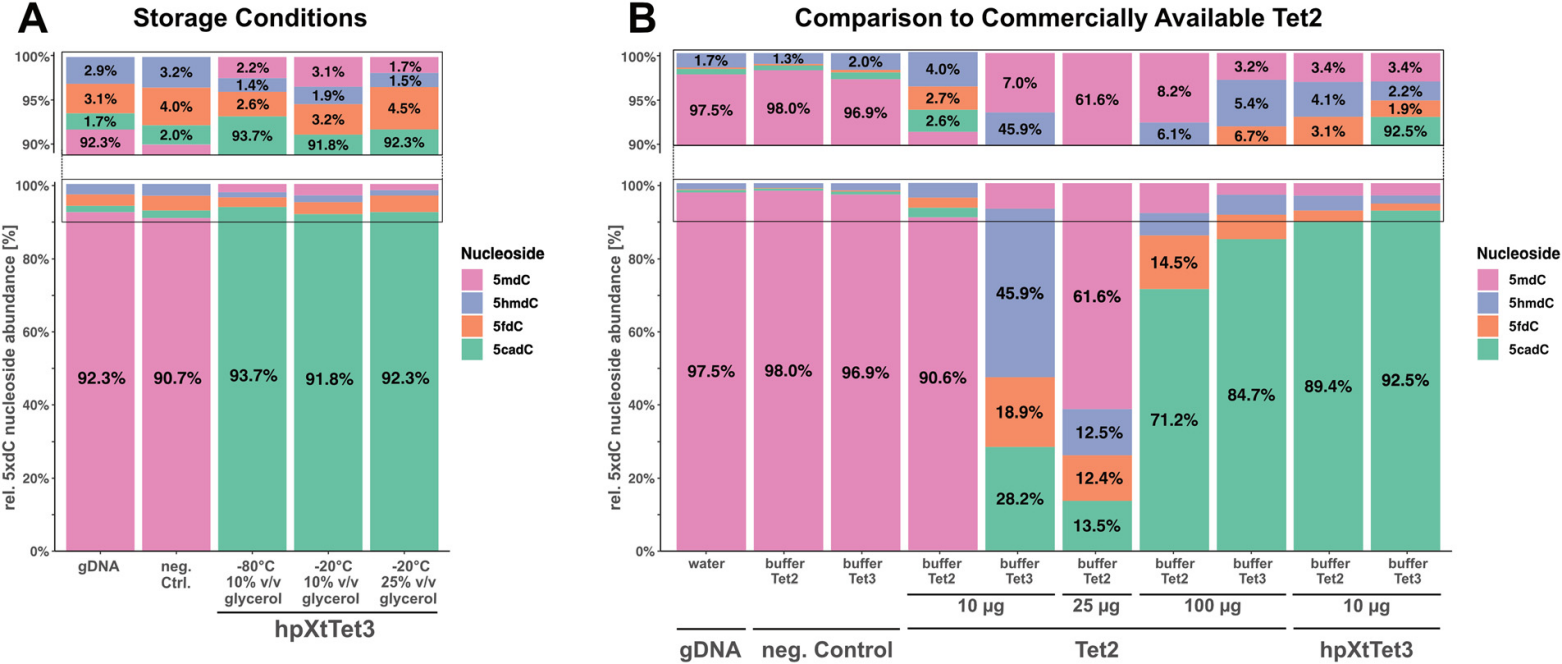
Impact of enzyme storage on oxidation activity and comparison with commercial Tet2. (A) Comparison of storage temperature (−20 °C *vs.* −80 °C) and glycerol concentration (10% *vs.* 25% v/v) in the storage buffer. (B) Direct comparison of 5mdC oxidation in 1 μg gDNA by Tet2 and hpXtTet3 in hpTet3- and Tet2-reaction buffer.

In addition, we tested storage conditions such as temperature (−80 °C *vs.* −20 °C) as well as storage buffer composition (10% *vs.* 25% v/v glycerol) using hpXtTet3, the slightly least stable of the three homologs. We observed that neither storage temperature nor the change in glycerol concentration impacted the oxidation activity of gDNA (Fig. 5A). However, for this experiment, we used gDNA from HEK293T cells of an earlier passage (passage 8) rather than in the above-mentioned experiments (passage 14) and detected that there are different levels of modified dC for this gDNA (Table S4, ESI†). It is known that in early passages, the characteristics of HEK293T cells more closely resemble those in the initial immortalization state, which also includes higher levels of epigenetic markers.^19,20^

Next, we attempted to compare the new hpXtTet3 with Tet2, which is currently in commercial use. We compared the activity of 10 μg Tet2 or hpXtTet3 on 1 μg gDNA, either using the reaction conditions described here or with supplied reaction buffers for Tet2. We found that hpXtTet3 showed conversion yields of 92.5% for 5mdC to 5cacdC, while for Tet2, the conversion efficiency was only 28% under the reaction conditions we used (Fig. 5). We next increased the amount of Tet2 by approximately 10-fold, and then Tet2 also reached conversion yields of 85% (Fig. 5B). Both enzymes showed higher oxidation activity under the buffer conditions described herein. We finally used 200 ng gDNA to test the activity of both enzymes, with 100 μg Tet2 or either 2 μg or 10 μg hpTet3 (Fig. S6, ESI†). In this setting, the conversion to 5cacdC by Tet2 reached almost 90%.

## Conclusions

Herein, we describe the development and investigation of three new high-performance Tet enzymes based on the sequences of Tet3. We found that all three hpTet3 enzymes efficiently oxidize 5mdC to 5cadC, with yields >97%. Under our reaction conditions, the tested Tet2 showed significantly lower 5mdC oxidation activity even when using 10 times higher enzyme concentration compared to hpXtTet3. As side products in the enzymatic 5mdC oxidation reaction, small amounts of 8oxodG and 5hmdU are formed, at levels that, in particular for 5hmdU, cannot be ignored in the context of TAB-seq and TAPS, which rely on the readout of 5mdC as dU or DHU, respectively. For other sequencing protocols, this side oxidation plays only a minor role (if at all) because the oxidation product 5hmdU codes in a manner similar to that of the starting material dT. The amount of 8-oxodG does not significantly increase, which is an important finding. Given that Tet enzymes utilize Fe^2+^ and an α-KG to generate a highly reactive Fe(4)=O species for the nucleobase oxidation, the small amounts of oxidation side products with highly shortened proteins are a surprise. These results, together with the high 5cadC yields, will assist in facilitating Tet3-based sequencing.

## Author contributions

ÖS and TW conducted experiments and analysed the data; HS purified mouse hpTet3; SSchn cloned and purified hpHsTet3 and hpXtTet3; and TC and SSchn supervised and planned the project, analysed the data, and wrote the first draft of the manuscript. All authors discussed the results and edited the final manuscript version.

## Data availability

The data supporting this article have been included as part of the ESI.†

## Conflicts of interest

There are no conflicts to declare.

## Supplementary Material

CB-OLF-D4CB00315B-s001

## References

[cit1] Tahiliani M., Koh K. P., Shen Y., Pastor W. A., Bandukwala H., Brudno Y., Agarwal S., Iyer L. M., Liu D. R., Aravind L., Rao A. (2009). Conversion of 5-methylcytosine to 5-hydroxymethylcytosine in mammalian DNA by MLL Partner TET1. Science.

[cit2] Globisch D., Munzel M., Muller M., Michalakis S., Wagner M., Koch S., Bruckl T., Biel M., Carell T. (2010). Tissue distribution of 5-hydroxymethylcytosine and search for active demethylation intermediates. PLoS One.

[cit3] Ito S., Shen L., Dai Q., Wu S. C., Collins L. B., Swenberg J. A., He C., Zhang Y. (2011). Tet proteins can convert 5-methylcytosine to 5-formylcytosine and 5-carboxylcytosine. Science.

[cit4] Su M., Kirchner A., Stazzoni S., Muller M., Wagner M., Schroder A., Carell T. (2016). 5-Formylcytosine could be a semipermanent base in specific genome sites. Angew. Chem., Int. Ed..

[cit5] Booth M. J., Ost T. W. B., Beraldi D., Bell N. M., Branco M. R., Reik W., Balasubramanian S. (2013). Oxidative bisulfite sequencing of 5-methylcytosine and 5-hydroxymethylcytosine. Nat. Protoc..

[cit6] Sahin H., Salehi R., Islam S., Müller M., Giehr P., Carell T. (2024). Robust bisulfite-free single-molecule real-time sequencing of methyldeoxycytidine based on a novel hpTet3 enzyme. Angew. Chem., Int. Ed..

[cit7] Vaisvila R., Ponnaluri V. K. C., Sun Z., Langhorst B. W., Saleh L., Guan S., Dai N., Campbell M. A., Sexton B. S., Marks K., Samaranayake M., Samuelson J. C., Church H. E., Tamanaha E., Corrêa I. R., Pradhan S., Dimalanta E. T., Evans T. C., Williams L., Davis T. B. (2021). Enzymatic methyl sequencing detects DNA methylation at single-base resolution from picograms of DNA. Genome Res..

[cit8] Frommer M., McDonald L. E., Millar D. S., Collis C. M., Watt F., Grigg G. W., Molloy P. L., Paul C. L. (1992). A genomic sequencing protocol that yields a positive display of 5-methylcytosine residues in individual DNA strands. Proc. Natl. Acad. Sci. U. S. A..

[cit9] Wang T., Fowler J. M., Liu L., Loo C. E., Luo M., Schutsky E. K., Berríos K. N., DeNizio J. E., Dvorak A., Downey N., Montermoso S., Pingul B. Y., Nasrallah M., Gosal W. S., Wu H., Kohli R. M. (2023). Direct enzymatic sequencing of 5-methylcytosine at single-base resolution. Nat. Chem. Biol..

[cit10] Liu Y., Siejka-Zielińska P., Velikova G., Bi Y., Yuan F., Tomkova M., Bai C., Chen L., Schuster-Böckler B., Song C.-X. (2019). Bisulfite-free direct detection of 5-methylcytosine and 5-hydroxymethylcytosine at base resolution. Nat. Biotechnol..

[cit11] Yu M., Hon Gary C., Szulwach Keith E., Song C.-X., Zhang L., Kim A., Li X., Dai Q., Shen Y., Park B., Min J.-H., Jin P., Ren B., He C. (2012). Base-resolution analysis of 5-hydroxymethylcytosine in the mammalian genome. Cell.

[cit12] Liu Y., Hu Z., Cheng J., Siejka-Zielińska P., Chen J., Inoue M., Ahmed A. A., Song C.-X. (2021). Subtraction-free and bisulfite-free specific sequencing of 5-methylcytosine and its oxidized derivatives at base resolution. Nat. Commun..

[cit13] Schutsky E. K., DeNizio J. E., Hu P., Liu M. Y., Nabel C. S., Fabyanic E. B., Hwang Y., Bushman F. D., Wu H., Kohli R. M. (2018). Nondestructive, base-resolution sequencing of 5-hydroxymethylcytosine using a DNA deaminase. Nat. Biotechnol..

[cit14] Carpenter M. A., Li M., Rathore A., Lackey L., Law E. K., Land A. M., Leonard B., Shandilya S. M., Bohn M. F., Schiffer C. A., Brown W. L., Harris R. S. (2012). Methylcytosine and normal cytosine deamination by the foreign DNA restriction enzyme APOBEC3A. J. Biol. Chem..

[cit15] Hu L., Li Z., Cheng J., Rao Q., Gong W., Liu M., Shi Y. G., Zhu J., Wang P., Xu Y. (2013). Crystal structure of TET2-DNA complex: insight into TET-mediated 5mC oxidation. Cell.

[cit16] Vogl J., Pritzkow W. (2010). Isotope dilution mass spectrometry - a primary method of measurement and its role for RM certification. Mapan.

[cit17] Kirouac K. N., Ling H. (2011). Unique active site promotes error-free replication opposite an 8-oxo-guanine lesion by human DNA polymerase iota. Proc. Natl. Acad. Sci. U. S. A..

[cit18] Hahm J. Y., Park J., Jang E. S., Chi S. W. (2022). 8-Oxoguanine: from oxidative damage to epigenetic and epitranscriptional modification. Exp. Mol. Med..

[cit19] Lund R. J., Narva E., Lahesmaa R. (2012). Genetic and epigenetic stability of human pluripotent stem cells. Nat. Rev. Genet..

[cit20] Razin A. R., Arthur D. (1980). DNA methylation and gene function. Science.

