# Supplementary material for: Novel Tet3 enzymes for next-generation epigenetic sequencing
Source: RSC Chem Biol. 2025 Mar 10;6(5):731–6. doi: 10.1039/d4cb00315b (PMC11915426; doi:10.1039/d4cb00315b)
Supplement: CB-006-D4CB00315B-s001 [file CB-006-D4CB00315B-s001.pdf]

This journal is © The Royal Society of Chemistry 2025

Supporting Information

Novel Tet3 enzymes for next generation epigenetic sequencing

Özge Simsir,* Tobias Walter,* Hanife Sahin, Thomas Carell§ and Sabine Schneider§

Department of Chemistry, Institute for Chemical Epigenetics, Ludwig-Maximilians Universität Munich, Butenandtstr. 5-13, 81377 Munich, Germany

MATERIAL AND METHODS

Cloning of hpHsTet3 cd and hpXtTet3

The codon optimized gene sequences (Azenta Life Sciences) encoding for the catalytic domains of Tet3 from *Homo sapiens* (Uniprot No. O43151, aa 824-1726, Δ1180-1635, hpHsTet3) and *Xenopus tropicalis* (Uniprot No. A0JP82, aa 953-1833, Δ1309-1742, hpXtTet3), with the low complexity insert deleted and replaced with a GS linker (Supporting Table 1, Supporting Figure 1) similar to the previously reported work on human Tet2[1], in frame with a N-terminal Strep-tag and Precission protease cleavage site, were cloned into pET28a using the NEB Hifi Assembly kit (New England Biolabs, Cat. No. E5520S). The identity of the sequences was confirmed by Sanger sequencing (Azenta Life Sciences). For protein expression the plasmids were transformed in *Escherichia coli* T7express (New England Biolabs, Cat. No. C2566H).

Protein expression and purification

For protein expression, an overnight culture of cells containing the respect expression plasmids (LB medium supplemented with 50 µg/ml kanamycin) was diluted 1:100 in 2YT medium (supplemented with 50 µg/ml kanamycin) and incubated at 37 °C, 180 rpm until an OD_{600} of ~0.8 was reached. Cells were cooled down on wet ice for 20 min and protein expression was induced with 0.5 mM isopropyl-β-D-1-thiogalactopyranoside (IPTG) and incubated overnight at 18°C. Cells were subsequently harvested by centrifugation (4000 × g; 20 min, RT) and the cell pellets stored at -20°C.

The cell pellet obtained from a 2-liter expression culture was resuspended in ~20 ml lysis buffer (50 mM Hepes, 500 mM NaCl, 10% v/v glycerol, 10 µM $ZnCl_2$ mM, 0.5 mM tris(2-chlorehyl)phosphat (TCEP), pH 6.8) supplemented with DNase I (AppliChem, Cat. No. A3778) and cOmplete™ protease inhibitors (Roche,

Cat. No. 1169749800). Cells were lysed by homogenization using an EmulsiFlexC5 (Avestin Inc.) and cleared lysate was obtained by centrifugation (20,000 × g; 20 min; 4 °C). Nucleic acids were removed from the lysate by adding dropwise polyethylenimine (8% w/v; Merck Cat. No. 408727) to a final concentration of 0.5% w/v, followed by a second centrifugation step (20,000 × g; 20 min; 4 °C).

The cleared lysate was filtered and loaded onto a 1 ml StrepTrap XT column (IBA BioSciences, Cat. No. 2-5024-001) attached to an ÄktaGo FPLC (Cytiva) at 8°C, the column washed with wash buffer (50 mM Hepes, 1.5 M NaCl, 10% v/v glycerol, 0.5 mM TCEP, pH 6.8) and the protein finally eluted with elution buffer (50 mM Hepes, 100 mM NaCl, 0.5 mM TCEP, 10% v/v glycerol, 50 mM biotin, pH 7.2). protein-containing fractions where diluted 1:10 in low-salt buffer (50 mM Hepes, pH 6.8, 0.5 mM TCEP, 10% v/v glycerol) and loaded onto a 1 ml HiTrap CaptoS column on an ÄktaGo FPLC (Cytiva) and eluted with a salt gradient (50mM Hepes pH 6.8, 1.5 M NaCl, 0.5 mM TCEP, 10% v/v glycerol). Fractions containing pure protein were pooled and buffer exchanged to storage buffer (25 mM Hepes pH 6.8, 150 mM NaCl, 10 % glycerol, 2 mM TCEP, pH 6.8), concentrated to 1 mg/ml using centrifugal filter devices (Amicon, Merck) aliquoted, flash-frozen in liquid nitrogen and stored at −80 °C. For investigation of the different storage conditions, the protein from one purification was either frozen in liquid nitrogen and stored at -20°C or -80°C, or supplemented with glycerol to a final concentration of 25% v/v and directly placed at -20°C.

The *Mus musculus* hpTet3 (hpMmTet3) (Uniprot No. A0A5K1VVP6) was expressed and purified as previously reported.[2]

Protein stability

Protein stability was analyzed using a Tycho™ NT.6 (Nanotemper Technologies) according to the manufactures protocol. Approximately 10 µl of a protein solution (0.3-0.5 mg/ml in 50 mM Hepes, 150 mM NaCl, 10% v/v glycerol) was applied in capillary. Than the tryptophan (and tyrosine) fluorescence at 330 nm and 350 nm over a temperature gradient (35°C-95°C) was determined. The 350 nm /330 nm ratio is measure for a spectral shift in the fluorescence and the emission profile from which the inflection temperature can be derived.

Sequence conservation mapping

For mapping the sequence conservation on the surface of the Alphafold model of human Tet3 ConSurf[3] was used with the following settings: Homologs were collected from the UNIREF90 database, with the HMMER search algorithm with an E-value cutoff of 0.0001 and homologues thresholds: hit cutoff is 97% (This is the maximal sequence identity between homologues); maximal number of final homologues =150; Maximal overlap between homologues is 10% (If overlap between two homologues exceeds 10%, the highest scoring homologue is chosen). Coverage is 60% (This is the minimal percentage of the query sequence covered by the homologue). Minimal sequence identity with the query sequence is 50%. The multiple sequence alignment was built using MAFFT and the conservation scores were calculated with the Bayesian method.
All structural figures were prepared with PyMol 2.4 (Schrodinger LLC).

HEK293T cell culture and gDNA extraction

Human embryonic kidney 293T (HEK293T; ATCC Cat. No. CRL-11268) cells were cultured in Dulbecco's Modified Eagle Medium (DMEM) High Glucose (PAN Biotech, Cat. No. P04-03590) supplemented with 10% v/v FBS (Sigma-Aldrich, Cat. No. F7524), and 1% Penicilin-Streptomycin (10,000 U/mL, Thermo Fisher Scientific, Cat. No. 15140122). Cultures were maintained at 37°C and 5% CO_2 and passaged every second day. Genomic DNA isolation was done from pelleted cells using Monarch® gDNA Purification Kit (New England Biolabs GmBH, Cat. No. T3010S) according to the manufacturer's protocol.

Oxidation reactions using hpTet3cd on synthetic DNA and HEK293T genomic DNA

Reactions were performed as previously reported.[2] In brief, 3 µM synthetic 5mdC containing 35mer DNA oligonucleotide (5′-CTATACCTCCTCAACTT **^{5}mC** GATCACCGTCTCCGGCG-3′; 3′-GATATGGAGGAGTTGAA**G**CTAGTGGCAGAGGCCGC-5′; Sigma-Aldrich) were incubated with 2 µM (5 µg) of the respective enzymes. Alternatively, 1 µg of freshly extracted human gDNA was incubated with 4 µM (10 µg) of each enzyme. To analyze the potential impact of enzyme storage 4 µM (10 µg) hpXtTet3, stored in different conditions was added: (1) storage buffer containing 10%v/v glycerol, direct freezing in liquid nitrogen, -20°C; (2) storage buffer containing 10%v/v glycerol, direct freezing in liquid nitrogen -80°C; (3) storage buffer containing 25%v/v glycerol, placed at -20°C without prior freezing in liquid nitrogen.

The oxidation reaction was carried out in a final volume of 50 µl in 50 mM HEPES pH 7.4., 50 mM NaCl, 1 mM α-ketoglutarate, 2 mM ascorbic acid, 1.2 mM ATP, 105 µM $Fe(NH_4)_2(SO_4)_2$ and 2.5 mM DTT. The stock solutions for ascorbic acid and $Fe(NH_4)_2(SO_4)_2$ were prepared fresh and $Fe(NH_4)_2(SO_4)_2$ was added last and directly before the addition of enzymes to the reaction in order to prevent the oxidation to Fe(III). All reactions were incubated at 37°C for 1 h at 500 rpm in a thermomixer (Eppendorf).

Following the oxidation reaction, 0.8 U of Proteinase K (New England Biolabs GmBH, Cat. No. P8107S) and SDS to a final concentration of 0.05% (w/v) were added to each sample and incubated at 50°C for 1 h for digestion of the hpTet3 enzymes, followed by heat-inactivation of Proteinase K (95°C, 10 min). The oxidized gDNA-samples were purified by 1.8x AMPure XP Beads (Beckman Coulter, Cat. No. A63882) and samples containing the synthetic dsDNA were purified using the Monarch® PCR&DNA Clean Up Kit (5 µg) (New England Biolabs GmBH, Cat. No. T1030L), according to the manufacturer's protocol. This was followed by DNA quantification by the Qubit™ dsDNA HS Assay (Invitrogen™, Cat. No. Q32854).

Following the purification, oxidized DNA samples were digested to single nucleosides by using a pre-mixed nucleoside digestion mix (New England Biolabs GmBH, Cat. No. M0649S) according to manufacturer's protocol. To remove further impurities from buffer components, prior to MS analysis, samples were filtered using the AcroPrep Advance 96-well Supor filter plate, 0.2 µm (Pall Life Sciences, Cytiva, Cat. No. 8019).

Comparative analysis of 5mdC oxidation in gDNA by hpXtTet3 and Tet2

For the comparison commercially available Tet2 (NEB Next® Enzymatic Methyl-seq Kit, Cat. No. E7120S) was used. Quantification of Tet2 was done via UV/Vis absorption spectroscopy as well as using SDS-PAGE analysis.

Since the Tet2-buffer composition is not known, Tet2 was diluted by the factors of 5 and 10 in hpTet3 storage buffer and the hpTet3 storage buffer was used as blank. Protein concentration was calculated as done for hpXtTet3, using at a millimolar absorbance of A_{280} = 1 corresponding to 1 mg/ml protein. To minimize impact of the unknown Tet2 storage buffer composition, we only used the 1:10 dilutions. we calculated the average concentration of Tet2 (1 in 10) = 0.25ug/ul and Tet2 (1 in 5) = 0.57ug/ul. To minimize impact of the unknown Tet2 storage buffer composition, we only used the 1 in 10 dilutions. For further comparison 1 µg of the respect proteins as determined spectroscopically described above were

analysed by SDS-PAGE, showing that both enzymes run at approximately the same size, with bands of comparable intensity following staining (Supporting Fig. S5).

For the oxidation reactions 1 µg of freshly extracted human gDNA was used and incubated with 10 µg hpXtTet3 or 10 µg, 25 µg and 100 µg Tet2 under hpTet3 and Tet2 reaction conditions.

In the manufacturers protocol, 4 µl Tet2 (~100 µg Tet2) are used to oxidise 200 ng genomic DNA. Therefore, we also incubated 200 ng gDNA either with 4 µl Tet2 or 2 µg hpXtTet3 under hpTet3 reaction conditions.

Following the oxidation reaction, samples with Tet3-buffer were subjected to a further incubation with 0.8 U of Proteinase K (New England Biolabs GmBH, Cat. No. P8107S) and SDS to a final concentration of 0.05% (w/v) at 50°C for 1 h to digest the enzymes, followed by a heat-inactivation of Proteinase K (95°C, 10 min). Alternatively, samples prepared according to the Tet2-protocol were incubated further at 37°C for 30 min after addition of the stop solution. DNA purification, single-nucleoside digestion and MS-sample preparation was done as stated above.

Analysis of conversion rates using UHPLC-MS/MS

Oxidation efficiency was determined by quantitative UHPLC-MS/MS (Triple Quadrupole LC/MS) analysis of digested DNA/ODN samples using an Agilent 1290 Infinity II UHPLC system equipped with a variable wavelength detector and an Agilent Technologies 6490 triple quadrupole mass spectrometer (ESI-MS). Chromatography was performed with an InfinityLab Poroshell 120 SB-C18 column (Agilent, 2.7 µm, 2.1 mm × 150 mm) at 35 °C using a gradient of water and MeCN, each containing 0.0075% (v/v) formic acid, at a flow rate of 0.35 mL/min: 0–4.0 min: 0%→3.5% (v/v) MeCN; 4.0–7.0 min: 3.5%→5.0% MeCN; 7.0–7.5 min: 5.0%→80% MeCN; 7.5–9.5 min: 80% MeCN; 9.5–10.0 min: 80→0% MeCN; 10.0–13.0 min: 0% MeCN. The autosampler was cooled to 4 °C and the injection volume was set to 10 µL for all samples. The nucleosides of interest were quantified using an adjusted version of the previously reported "stable isotope dilution technique".[4]

The source-dependent parameters were as follows: gas temperature 120 °C, gas flow 11 L/min (N_2), nebulizer 60 psi, sheath gas temperature 280 °C, sheath gas flow 11 L/min (N_2), capillary voltage 3000 V in positive ion mode, nozzle voltage 500 V, high-pressure RF at 150 V and low-pressure RF at 60 V. The compound-dependent parameters are summarized Supporting Table S3. For all compounds, the

fragmentor voltage was set to 380 V and the Electron multiplier voltage was set to 500 V (positive mode). Each sample was spiked with 1 µL of 0.5 µM (0.5 pmol) of a stable isotope-labeled internal standard (ISTD) mix containing the isotope standards stated in Supporting Table S3.

The sample data were analyzed by Quantitative MassHunter Software (Agilent, Version B07.01) using the built-in calibration function. The results of Quantification are summarized in Supporting Table S4.

SUPPORTING TABLES

Supporting Table S1. Protein sequences of the hpTet3 enzymes. Highlighted in blue is the GS-linker replacing the low complexity insert (LCI). The Strep-tag II sequence is marked in bold, underlined are the Human Rhino Virus (HRV) 3C protease (hpHsTet3; hpXtTet3) and Tabacco Etch Virus (TEV) protease (hpMmTet3) cleavage sites, respectively.

Name	Amino acid sequence	Ref.
hpHsTet3<br>Uniprot No.:<br>O43151	**MASWSHPQFEK**SGGGGGGALEVLFQGPEFPTCDCVEQIVEKDEGPYYTHLGSGPTVASIR<br>ELMEERYGEKGKAIRIEKVIYTGKEGKSSRGCPIAKWVIRRHTLEEKLLCLVRHRAGHHC<br>QNAVIVILILAWEGIPRSLGDTLYQELTDTLRKYGNPTSRRCGLNDDRTCACQGKDPNTC<br>GASFSFGCSWSMYFNGCKYARSKTPRKFRLAGDNPKEEEVLRKSFQDLATEVAPLYKRLA<br>PQAYQNQVTNEEIAIDCRLGLKEGRPFAGVTACMDFCAHAHKDQHNLYNGCTVVCTLTKE<br>DNRCVGKIPEDEQLHVLPLYKMANTDEFGSEENQNAKVGSGAIQVLTAFPREVRRLPEPA<br>KSCRQRQLEARKAAAEKKKIQKEK**GGGGSGGGGSGGGG**EEELWSDSEHNFLDENIGGVAV<br>APAHGSILIECARRELHATTPLKKPNRCHPTRISLVFYQHKNLNQPNHGLALWEAKMKQL<br>AERARARQEE	This work
hpXtTet3<br>Uniprot No.:<br>A0JP82	**MASWSHPQFEK**SGGGGGGALEVLFQGPEFPTCDCVEQINEKDEGPYYTHLGSGPTVASIR<br>ELMEERFGQKGDAIRIEKVIYTGKEGKSSRGCPIAKWVIRRQSEDEKLMCLVRQRAGHHC<br>ENAVIIIILIMAWEGIPRSLGDSLYNDITETITKYGNPTSRRCGLNDDRTCACQGKDPNTC<br>GASFSFGCSWSMYFNGCKYARSKTPRKFRLIGENPKEEDGLKDNFQNLATKVAPVYKMLA<br>PQAYQNQVNNEDIAIDCRLGLKEGRPFSGVTACMDFCAHAHKDQHNLYNGCTVVCTLTKE<br>DNRMIGRVAEDEQLHVLPLYKVSTTDEFGSEEGQLEKIKKGGIHVLSSFPREVRKLSEPA<br>KSCRQRQLEAKKAAAEKKKLQKEK**GGGGSGGGGSGGGG**DEEIWSDSEHNFLDENIGGVAV<br>APGHGSILIECARRELHATTPLKKPNRCHPTRISLVFFQHKNLNQPNHGLALWEAKMKQL<br>AERARAREEE	This work
hpMmTet3<br>Uniprot No.:<br>A0A5K1VVP6	**MASWSHPQFEK**SGGGGGENLYFQGSEFPTCDCVEQIVEKDEGPYYTHLGSGPTVASIREL<br>MEDRYGEKGKAIRIEKVIYTGKEGKSSRGCPIAKWVIRRHTLEEKLLCLVRHRAGHHCQN<br>AVIVILILAWEGIPRSLGDTLYQELTDTLRKYGNPTSRRCGLNDDRTCACQGKDPNTCGA<br>SFSFGCSWSMYFNGCKYARSKTPRKFRLTGDNPKEEEVLRNSFQDLATEVAPLYKRLAPQ<br>AYQNQVTNEDVAIDCRLGLKEGRPFSGVTACMDFCAHAHKDQHNLYNGCTVVCTLTKEDN<br>RCVGQIPEDEQLHVLPLYKMASTDEFGSEENQNAKVSSGAIQVLTAFPREVRRLPEPAKS<br>CRQRQLEARKAAAEKKK**GGGGSGGGGSGGGGES**ELWSDSEHNFLDENIGGVAVAPAHCSI<br>LIECARRELHATTPLKKPNRCHPTRISLVFYQHKNLNQPNHGLALWEAKMKQLAERARQR<br>QEEAARLG	Ref [2]

Supporting Table S2. Sequence identity (id.) and similarity (sim.) of the catalytic domains of Tet3 homologs investigated in this study, and the respect Tet2 homologs, using EMBOSS Needle Pairwise Sequence Alignment (PSA) (EMBL-EBI https://www.ebi.ac.uk/jdispatcher/psa/emboss_needle). N-term and C-term corresponds to the sequence preceding and following the low complexity insert (LCI). The overall sequence identity/similarity of the catalytic domains were calculated excluding the LCI., HsTet2 Uniprot No. Q6N021, MmTet2 Uniprot No. Q4JK59, XtTet2 Uniprot No. F6XM2

<table>
<tr><th rowspan="3"></th><th colspan="2">hpHsTet3</th><th colspan="2">hpMmTet3</th><th colspan="2">hpXtTet3</th></tr>
<tr><td>Id./sim.</td><td>Id./sim.</td><td>Id./sim.</td><td>Id./sim.</td><td>Id./sim.</td><td>Id./sim.</td></tr>
<tr><td>N-term</td><td>C-term</td><td>N-term</td><td>C-term</td><td>N-term</td><td>C-term</td></tr>
<tr><td rowspan="2">HsTet2</td><td>71.2/83.3</td><td>72.9/84.4</td><td rowspan="2" colspan="4"></td></tr>
<tr><td colspan="2">Overall: 71.6/83.5</td></tr>
<tr><td rowspan="2">hpHsTet3</td><td rowspan="2" colspan="2"></td><td>96.1/97.8</td><td>91.8/91.8</td><td>84.3/93.6</td><td>94.3/98.9</td></tr>
<tr><td colspan="2">Overall: 94.9/96.3</td><td colspan="2">Overall: 86.4/94.7</td></tr>
<tr><td rowspan="2">MmTet2</td><td rowspan="2" colspan="2"></td><td>57.7/68.4</td><td>55.8/64.6</td><td rowspan="2" colspan="2"></td></tr>
<tr><td colspan="2">Overall: 57.3/67.9</td></tr>
<tr><td rowspan="2">hpMmTet3</td><td rowspan="2" colspan="2"></td><td rowspan="2" colspan="2"></td><td>83.2/92.7</td><td>86.6/89.7</td></tr>
<tr><td colspan="2">Overall: 84.1/92.3</td></tr>
<tr><td rowspan="2">XtTet2</td><td rowspan="2" colspan="2"></td><td rowspan="2" colspan="2"></td><td>67.2/81.9</td><td>61.1/69.9</td></tr>
<tr><td colspan="2">Overall: 65.8/78.9</td></tr>
</table>

Supporting Table S3. Compound-dependent LC-MS/MS-parameters. CE: collision energy; CAV: collision cell accelerator voltage, t_R: retention time.

Compound	Precursor Ion [m/z]	MS1 Resolution	Product Ion [m/z]	MS2 Resolution	t_R [min]	Δt_R [min]	CE [V]	CAV [V]	Polarity
**[$^{15}N_5$]-8oxodG**	289.08	Wide	173.04	Unit	7.1	6	9	5	Positive
**8oxodG**	284.10	Wide	168.05	Unit	7.1	6	9	5	Positive
**[$^{15}N_2$-$^{13}C_5$]-dT**	250.10	Wide	129.10	Unit	6.8	3	3	5	Positive
**dT**	243.10	Wide	127.05	Unit	6.8	3	3	5	Positive
**[$^{15}N_5$-$^{13}C_{10}$]-dA**	267.10	Wide	146.10	Unit	6.7	4	12	5	Positive
**dA**	252.10	Wide	136.10	Unit	6.7	4	12	5	Positive
**[$^{15}N_2$]-fdC**	258.09	Wide	142.04	Unit	6.4	3	5	5	Positive
**fdC**	256.09	Wide	140.05	Unit	6.4	3	5	5	Positive
**[^{15}N5-$^{13}C_{10}$]-dG**	283.10	Wide	162.06	Unit	6.0	3	6	5	Positive
**dG**	268.00	Wide	152.10	Unit	6.0	3	6	5	Positive
**[D_2]-hmdU**	261.10	Wide	145.10	Unit	4.4	3	4	5	Positive
**hmdU**	259.10	Wide	143.10	Unit	4.4	3	4	5	Positive
**[D_3]-mdC**	245.13	Wide	129.09	Unit	4.2	3	4	5	Positive
**mdC**	242.11	Wide	126.07	Unit	4.2	3	4	5	Positive
**[$^{15}N_2$]-cadC**	274.08	Wide	158.03	Unit	4.1	3	6	5	Positive

**cadC**	272.09	Wide	156.04	Unit	4.1	3	6	5	Positive
**[D_2-$^{15}N_2$]-hmdC**	262.12	Wide	146.07	Unit	2.6	3	4	5	Positive
**hmdC**	258.11	Wide	142.06	Unit	2.6	3	4	5	Positive
**[$^{13}C_9$-$^{15}N_2$]-dC**	239.10	Wide	118.10	Unit	2.6	3	5	5	Positive
**dC**	228.10	Wide	112.10	Unit	2.6	3	5	5	Positive

Supporting Table S4. Abundance of modified cytidine nucleosides in HEK293T gDNA used for the evaluation of the performance of the hpTet3 enzymes. From the two biological replicates three technical replicates were analyzed. gDNA: isolated genomic DNA was digested to nucleoside level and the present nucleosides were quantified. Control: nucleoside abundance in gDNA following the incubation in the oxidation buffer in the absences of hpTet3-enzymes.

	**HEK293T-Passage 14 (Batch 1)**				**HEK293T-Passage 8 (Batch 1)**			
**Nucleoside**	**Control 1**	**Control 2**	**gDNA 1**	**gDNA 2**	**Control 1 (NEB Buf.)**	**Control 2 (Tet3 Buf.)**	**gDNA 1**	**gDNA2**
**mdC**	4.06% ± 0.12%	3.58% ± 0.79%	3.97% ± 0.22%	4.07% ± 0.25%	2.96% ± 0.32%	3.04% ± 0.13%	3.52% ± 0.19%	3.06% ± 0.35%
**hmdC**	0.02% ± 0.00%	0.03% ± 0.00%	0.02% ± 0.00%	0.03% ± 0.00%	0.08% ± 0.00%	0.07% ± 0.00%	0.15% ± 0.00%	0.07% ± 0.00%
**fdC**	0.05% ± 0.00%	0.03% ± 0.00%	0.10% ± 0.00%	0.02% ± 0.00%	0.12% ± 0.00%	0.09% ± 0.01%	0.15% ± 0.00%	0.09% ± 0.00%
**cadC**	0.00% ± 0.00%	0.01% ± 0.01%	0.01% ± 0.00%	0.00% ± 0.00%	0.06% ± 0.00%	0.04% ± 0.00%	0.09% ± 0.00%	0.04% ± 0.00%
**dC**	95.86% ± 1.98%	96.28% ± 3.99%	95.90% ± 7.8%	95.88% ± 3.04%	96.78% ± 10.43%	96.76% ± 5.84%	96.09% ± 7.09%	96.74% ± 9.78%

Supporting Table S5. Nucleoside abundance of hpTet3-treated dsODN and HEK293T gDNA. The mean data ± SE of three technical replicates are normalized to the dT amount in the sample and is displayed in the power of $\times 10^3$ for easier interpretation.

	**3 µM dsODN**					**1ug gDNA**				
**Nucleoside (mean)**	**hpHsTET3**	**hpMmTET3**	**hpXtTET3**	**Control**	**ODN (200 ng)**	**hpHsTET3**	**hpMmTET 3**	**hpXtTET3**	**Control**	**gDNA (150 ng)**
**mdC/dT [$\times 10^3$]**	3.550 ± 0.463	2.900 ± 0.361	2.488 ± 0.458	109.883 ± 4.981	98.148 ± 4.640	0.236 ± 0.043	0.251 ± 0.030	0.153 ± 0.036	23.976 ± 0.702	24.173 ± 1.336
**hmdC/dT [$\times 10^3$]**	7.678 ± 0.874	1.870 ± 0.079	2.014 ± 0.135	0.041 ± 0.009	0.009 ± 0.000	0.213 ± 0.035	0.222 ± 0.029	0.124 ± 0.027	0.135 ± 0.003	0.152 ± 0.006
**fdC/dT [$\times 10^3$]**	31.124 ± 4.444	2.257 ± 0.185	2.953 ± 0.166	0.331 ± 0.023	0.111 ± 0.001	0.744 ± 0.033	0.786 ± 0.012	0.594 ± 0.019	0.322 ± 0.000	0.582 ± 0.004
**cadC/dT [$\times 10^3$]**	145.021 ± 17.858	176.999 ± 13.125	170.932 ± 3.733	0.083 ± 0.034	0.014 ± 0.002	43.569 ± 2.545	44.046 ± 4.080	42.602 ± 3.261	0.029 ± 0.002	0.065 ± 0.008

Supporting Table S6. 8-oxodG and hmdU abundance of hpXtTet3-treated HEK293T gDNA depending on the enzyme amount. The mean data ± SE of three technical replicates are normalized to the dT or dG amount in the sample and is displayed in the power of $\times 10^3$ for easier interpretation.

	Side Product Formation depending on Enzyme Amount (hpXtTet3)				
Nucleoside (mean)	10 µg	15 µg	20 µg	Neg. Control	DNA (150 ng)
8-oxodG/dT [$\times 10^3$]	0.197 ± 0.008	0.221 ± 0.011	0.222 ± 0.009	0.261 ± 0.013	0.054 ± 0.008
hmdU/dG [$\times 10^3$]	3.751 ± 0.513	2.809 ± 0.353	2.092 ± 0.018	0.162 ± 0.044	0.033 ± 0.004

Supporting Table S7. Nucleoside abundance of Tet-treated HEK293T gDNA. In these experiments, the activity of hpXtTet3 after different storage conditions. The mean data ± SE of three technical replicates are normalized to the dT amount (dG for hmdU) in the sample and is displayed in the power of $\times 10^3$ for easier interpretation.

	Storage conditions (hpXtTet3)				
Nucleoside (mean)	gDNA	Neg. Control	-80°C 10% Gly	-20°C 10% Gly	-20°C 25% Gly
mdC/dT [$\times 10^3$]	24.119 ± 6.921	23.302 ± 8.924	0.854 ± 0.112	1.21 ± 0.105	0.630 ± 0.022
hmdC/dT [$\times 10^3$]	0.745 ± 0.034	0.828 ± 0.037	0.538 ± 0.029	0.724 ± 0.029	0.542 ± 0.011
fdC/dT [$\times 10^3$]	0.808 ± 0.053	1.029 ± 0.062	0.996 ± 0.074	1.260 ± 0.157	1.673 ± 0.063
cadC/dT [$\times 10^3$]	0.450 ± 0.004	0.525 ± 0.010	35.601 ± 1.621	35.641 ± 5.41	34.249 ± 1.998
8-oxodG/dT [$\times 10^3$]	0.116 ± 0.016	0.213 ± 0.037	0.168 ± 0.010	0.180 ± 0.016	0.164 ± 0.014
hmdU/dG [$\times 10^3$]	1.007 ± 0.068	1.176 ± 0.047	3.573 ± 0.104	3.279 ± 0.448	2.648 ± 0.189

Supporting Table S8. Nucleoside abundance of Tet-treated HEK293T gDNA. In these experiments, the performance of hpXtTet3 compared to a commercially available Tet2 enzyme was examined. The mean data ± SE of three technical replicates are normalized to the dT amount (dG for hmdU) in the sample and is displayed in the power of $\times 10^3$ for easier interpretation.

	Comparison of hpXtTet3 and commercially available Tet2									
**Nucleoside (mean)**	**Neg. Ctrl. (Tet2 buffer)**	**Neg. Ctrl. (Tet3 buffer)**	**gDNA**	**10 µg Tet2 (Tet2 buffer)**	**10 µg Tet2 (Tet3 buffer)**	**25 µg Tet2 (Tet2 buffer)**	**100 µg Tet2 (Tet2 buffer)**	**100 µg Tet2 (Tet3 buffer)**	**10 µg hpXtTet3 (Tet2 buffer)**	**10 µg hpXtTet3 (Tet3 buffer)**
**mdC/dT [×10³]**	40.722 ± 3.783	36.901 ± 2.274	37.829 ± 3.384	9.580 ± 0.559	1.930 ± 0.292	1.763 ± 0.132	0.767 ± 0.003	1.185 ± 0.065	0.334 ± 0.003	1.131 ± 0.112
**hmdC/dT [×10³]**	0.535 ± 0.015	0.751 ± 0.008	0.654 ± 0.006	0.425 ± 0.004	12.716 ± 0.678	0.358 ± 0.001	0.575 ± 0.002	2.039 ± 0.349	0.398 ± 0.000	0.741 ± 0.021
**fdC/dT [×10³]**	0.072 ± 0.001	0.112 ± 0.007	0.070 ± 0.004	0.290 ± 0.015	5.241 ± 0.026	0.356 ± 0.012	1.364 ± 0.142	2.494 ± 0.378	0.305 ± 0.014	0.654 ± 0.025
**cadC/dT [×10³]**	0.235 ± 0.001	0.303 ± 0.002	0.247 ± 0.001	0.275 ± 0.018	7.821 ± 1.017	0.387 ± 0.010	6.696 ± 1.320	31.726 ± 5.872	8.745 ± 0.633	31.154 ± 1.028
**8-oxodG/dT [×10³]**	0.279 ± 0.004	0.423 ± 0.008	0.269 ± 0.007	0.244 ± 0.022	0.429 ± 0.014	0.227 ± 0.010	0.130 ± 0.002	0.254 ± 0.034	0.272 ± 0.021	0.413 ± 0.039
**hmdU/dG [×10³]**	0.928 ± 0.251	0.893 ± 0.042	0.979 ± 0.447	0.997 ± 0.176	1.049 ± 0.016	0.648 ± 0.039	1.245 ± 0.030	2.148 ± 0.326	1.363 ± 0.040	3.179 ± 0.094

Supporting Table S9. Nucleoside abundance of Tet-treated HEK293T gDNA. With 200 ng of gDNA material, the performance of hpXtTet3 compared to a commercially available Tet2 enzyme was examined using Tet3 buffer (see Supporting Figure S6). The mean data ± SE of three technical replicates are normalized to the dT amount (dG for hmdU) in the sample and is displayed in the power of $\times 10^3$ for easier interpretation.

	Comparison of hpXtTet3 and commercially available Tet2 in Tet3 Buffer and 200 ng gDNA				
**Nucleoside (mean)**	**gDNA**	**Neg. Control**	**Tet2 (100 µg)**	**hpXtTet3 (2 µg)**	**hpXtTet3 (10 µg)**
**mdC/dT [×10³]**	37.829 ± 3.384	36.807 ± 3.469	1.180 ± 0.252	1.088 ± 0.012	0.757 ± 0.103
**hmdC/dT [×10³]**	0.654 ± 0.006	0.971 ± 0.010	1.647 ± 0.198	0.731 ± 0.007	0.776 ± 0.006
**fdC/dT [×10³]**	0.070 ± 0.004	0.134 ± 0.004	1.425 ± 0.050	0.747 ± 0.013	1.337 ± 0.100
**cadC/dT [×10³]**	0.247 ± 0.001	0.405 ± 0.002	30.363 ± 2.053	34.409 ± 2.126	35.14 ± 1.282
**8-oxodG/dT [×10³]**	0.269 ± 0.007	0.540 ± 0.010	0.563 ± 0.159	0.418 ± 0.001	0.377 ± 0.004
**hmdU/dG [×10³]**	0.979 ± 0.447	0.956 ± 0.028	3.096 ± 0.470	3.416 ± 0.033	2.933 ± 0.055

SUPPORTING FIGURES

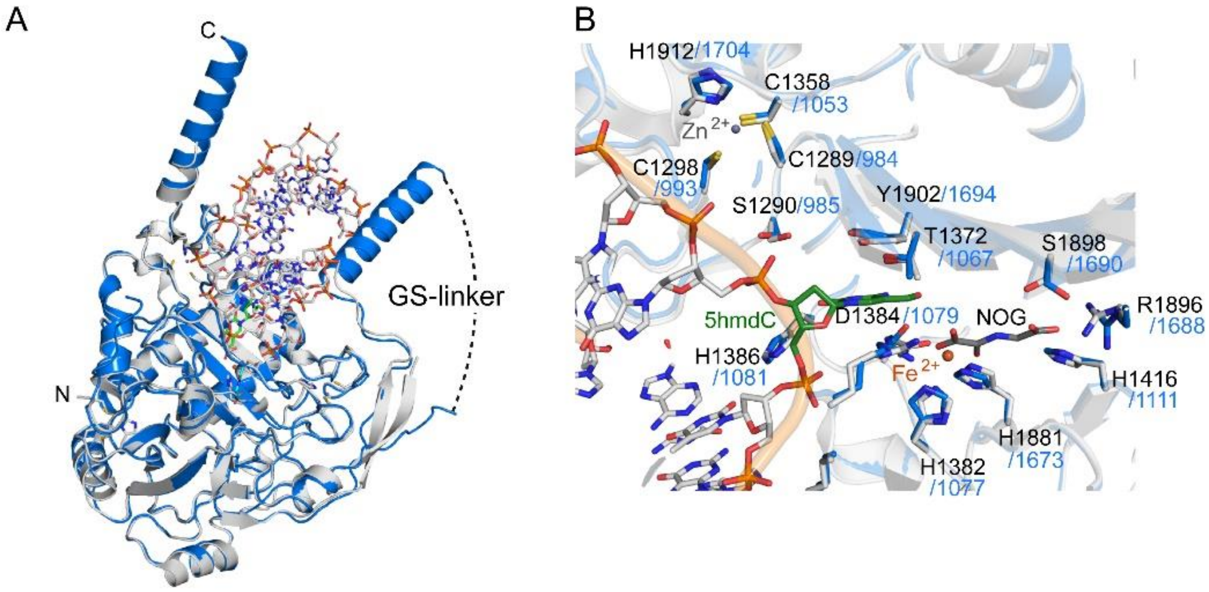

Supporting Figure S1. Structural comparison of *Homo sapiens* Tet2 with the AlphaFold prediction of *Homo sapiens* Tet3 (Uniprot No. O43151, AFDB: AF-O43151-F1-v4). A) Superposition of X-ray crystal structure of the catalytic domain of HsTet2 in complex with dsDNA containing 5hmdC (PDB code 5DEU, grey) and the Alphafold2 model of human Tet3 (blue). The location of the GS-linker that replaces the low complexity insert (LCI) is indicated as dashed line. B) Zoom in the active site: highlighted as stick representation are the active site residues that are lining the active site and coordinating the iron (shown as sphere), the N-oxalylglycine (dark grey), substituting the 2-oxoglutarate cofactor, as well as the 5hmdC (green) flipped-out of the dsDNA duplex and positioned within the catalytic centre. Numbering in grey and blue corresponds to HsTet2 and HsTet3, respectively.

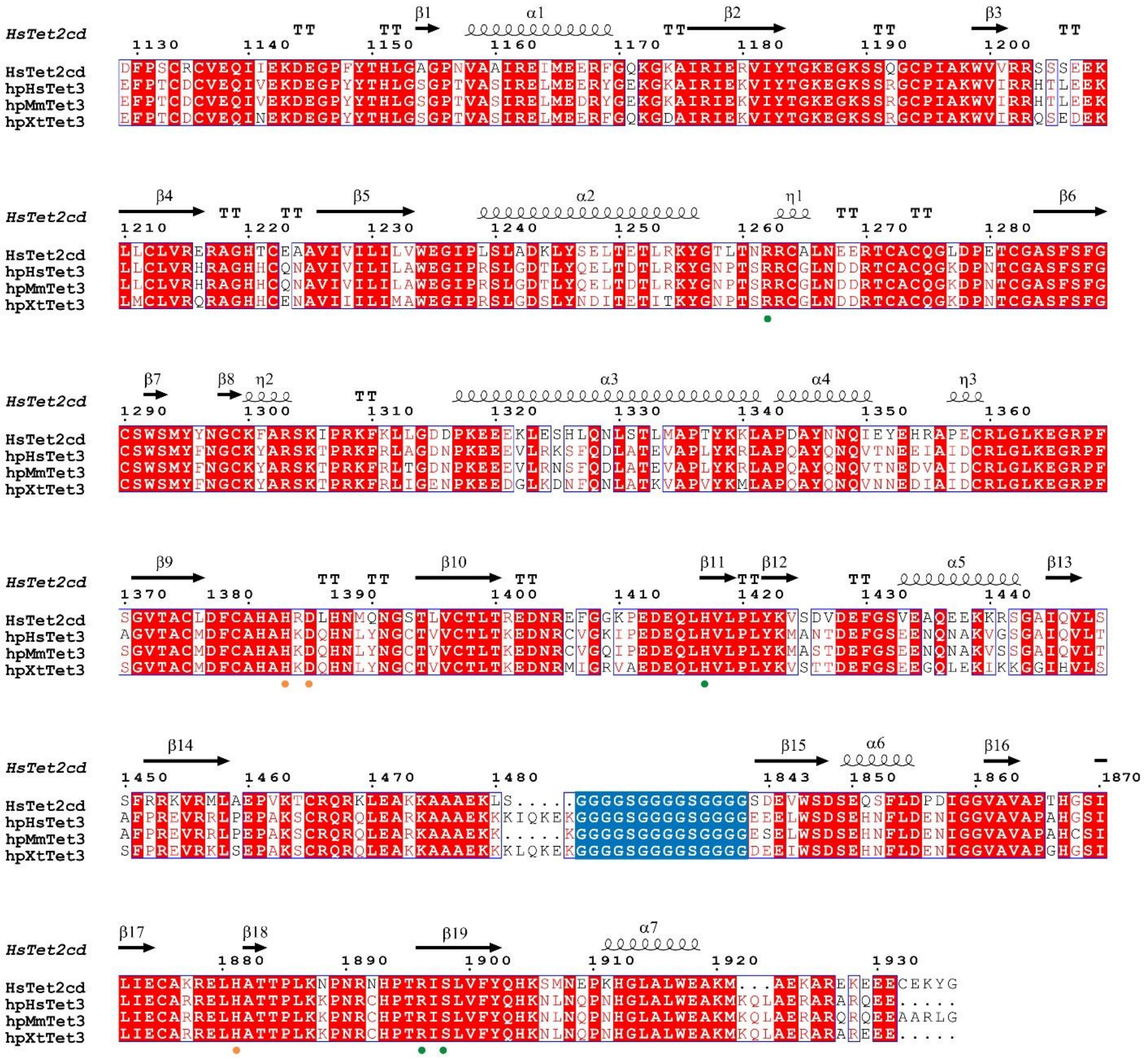

Supporting Figure S2: Sequence alignment of hpTet3 homologs and comparison with HsTet2. Secondary structure annotation and numbering corresponds to the crystallised construct of HsTet2 (PDB code 5DEU). Highlighted in blue is the GS-linker sequence that replaces the LCI. The orange and green dots mark the residues coordinating the iron and α-KG, respectively. Sequences were aligned using Clustal[5] and the alignment was annotated using ESPript 3.0.[6]

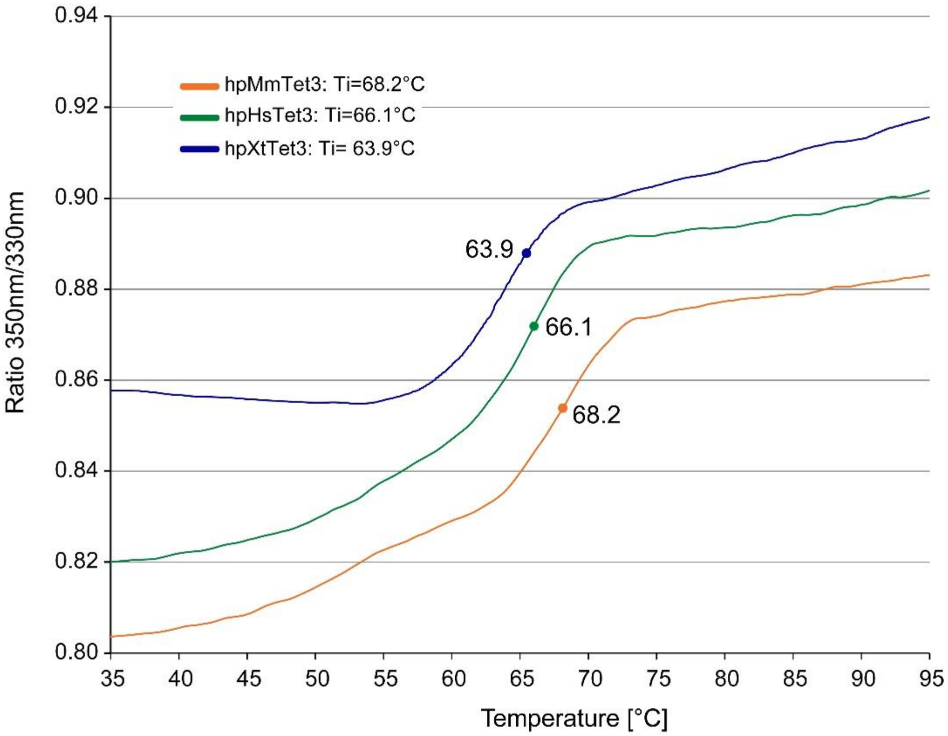

Supporting Figure S3. Stability of different Tet3cd homologs. Temperature dependent unfolding profile curves (fluorescence ratio 350nm/330nm) and inflection temperatures (T_i).

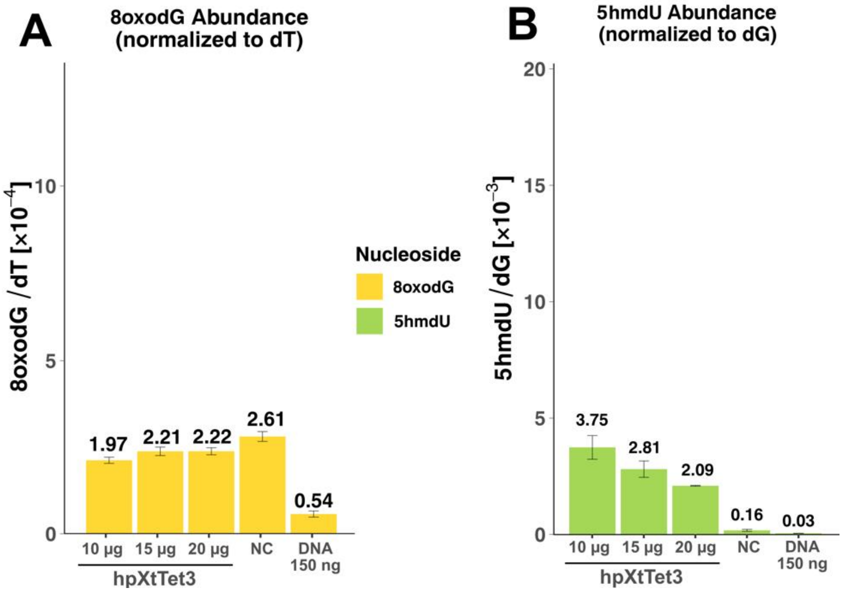

Supporting Figure S4. Analysis of the oxidation byproducts 8-oxodG (A) and 5hmdU (B) in hpXtTet3-treated HEK293T gDNA in relation to the enzyme amount.

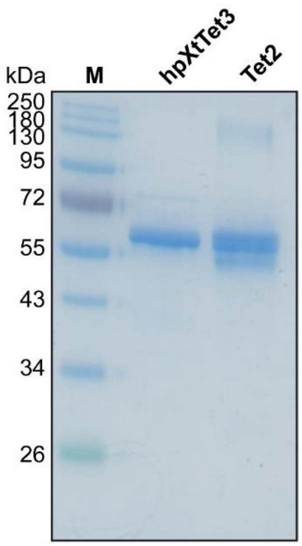

Supporting Figure S5. SDS-PAGE analysis of Tet2 and hpXtTet3 using a 12% SDS-PAGE gel. M= Marker; lane 1: hpXtTet3 (1 µg); lane 2: Tet2 (1 µg as determined using UV/Vis spectroscopy).

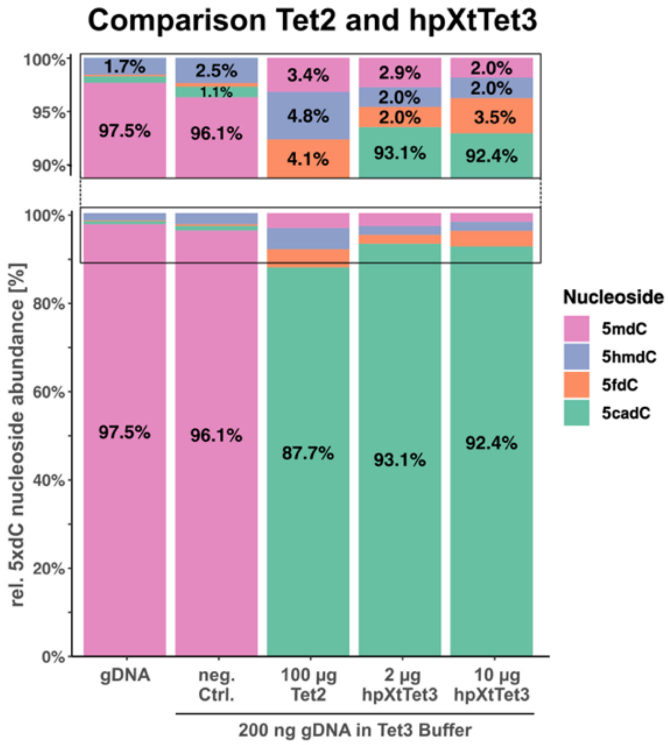

Supporting Figure S6. Oxidation of 5mdC in 200 ng genomic DNA by hpXtTet3 (2 µg and 10 µg) and Tet2 (4 µl ≈100 µg) in Tet3 buffer. According to the supplier 4 µl Tet2 are used to treat up to 200 ng gDNA.

REFERENCES

1. L. Hu, J. Lu, J. Cheng, Q. Rao, Z. Li, H. Hou, Z. Lou, L. Zhang, W. Li, W. Gong, M. Liu, C. Sun, X. Yin, J. Li, X. Tan, P. Wang, Y. Wang, D. Fang, Q. Cui, P. Yang, C. He, H. Jiang, C. Luo and Y. Xu, Structural insight into substrate preference for TET-mediated oxidation, *Nature*, 2015, **527**, 118-122.
2. H. Sahin, R. Salehi, S. Islam, M. Müller, P. Giehr and T. Carell, Robust bisulfite-free single-molecule real-time sequencing of methyldeoxycytidine based on a novel hpTet3 enzyme, *Angew. Chem. Int. Ed. Engl.*, 2024, **63**, e202418500.
3. B. Yariv, E. Yariv, A. Kessel, G. Masrati, A. B. Chorin, E. Martz, I. Mayrose, T. Pupko and N. Ben-Tal, Using evolutionary data to make sense of macromolecules with a "face-lifted" ConSurf, *Protein Sci.*, 2023, **32**, e4582.
4. F. R. Traube, S. Schiffers, K. Iwan, S. Kellner, F. Spada, M. Muller and T. Carell, Isotope-dilution mass spectrometry for exact quantification of noncanonical DNA nucleosides, *Nat. Protoc.*, 2019, **14**, 283-312.
5. J. D. Thompson, D. G. Higgins and T. J. Gibson, CLUSTAL W: improving the sensitivity of progressive multiple sequence alignment through sequence weighting, position-specific gap penalties and weight matrix choice, *Nucleic Acids Res.*, 1994, **22**, 4673-4680.
6. X. Robert and P. Gouet, Deciphering key features in protein structures with the new ENDscript server, *Nucleic Acids Res.*, 2014, **42**, W320-324.
